# Effectiveness of chest pain center accreditation on the hospital outcome of acute aortic dissection: a nationwide study in China

**DOI:** 10.1186/s40779-024-00565-0

**Published:** 2024-08-26

**Authors:** Li-Wei Liu, Yi-Kai Cui, Lin Zhang, Dai-Le Jia, Jing Wang, Jia-Wei Gu, Jin-Yan Zhang, Zhen Dong, Xue-Juan Jin, Xiao-Yi Zou, Guo-Li Sun, Yu-Xiang Dai, Ai-Jun Sun, Jun-Bo Ge

**Affiliations:** 1 Department of Cardiology, Zhongshan Hospital, Institutes of Biomedical Sciences, Fudan University, Shanghai Institute of Cardiovascular Diseases, Shanghai, 200032 China; 2 Institutes of Biomedical Sciences, Fudan University, Shanghai, 200032 China; 3 State Key Laboratory of Cardiology, Zhongshan Hospital, Fudan University, Shanghai, 200032 China; 4 Key Laboratory of Viral Heart Diseases, National Health Commission, Shanghai, 200032 China; 5 Department of Emergency and Critical Care Medicine, Jinshan Hospital of Fudan University, Shanghai, 201508 China; 6 Reproductive Medicine Center, Zhongshan Hospital, Fudan University, Shanghai, 200032 China; 7 Department of Cardiac Surgery, Zhongshan Hospital, Fudan University, Shanghai, 200032 China; 8 Department of Cardiology, Copenhagen University Hospital, 2100 Copenhagen, Denmark

**Keywords:** Acute aortic dissection (AAD), Chest pain center (CPC), Accreditation, In-hospital outcomes

## Abstract

**Background:**

The National Chest Pain Center Program (NCPCP) is a nationwide, quality enhancement program aimed at raising the standard of care for patients experiencing acute chest pain in China. The benefits of chest pain center (CPC) accreditation on acute coronary syndrome have been demonstrated. However, there is no evidence to indicate whether CPC accreditation improves outcomes for patients with acute aortic dissection (AAD).

**Methods:**

We conducted a retrospective observational study of patients with AAD from 1671 hospitals in China, using data from the NCPCP spanning the period from January 1, 2016 to December 31, 2022. The patients were divided into 2 groups: pre-accreditation and post-accreditation admissions. The outcomes examined included in-hospital mortality, misdiagnosis, and Stanford type A AAD surgery. Multivariate logistic regression was employed to explore the relationship between CPC accreditation and in-hospital outcomes. Furthermore, we stratified the hospitals based on their geographical location (Eastern/Central/Western regions) or administrative status (provincial/non-provincial capital areas) to assess the impact of CPC accreditation on AAD patients across various regions.

**Results:**

The analysis encompassed a total of 40,848 patients diagnosed with AAD. The post-accreditation group exhibited significantly lower rates of in-hospital mortality and misdiagnosis (12.1% vs. 16.3%, *P* < 0.001 and 2.9% vs. 5.4%, *P* < 0.001, respectively) as well as a notably higher rate of Stanford type A AAD surgery (61.1% vs. 42.1%, *P* < 0.001) compared with the pre-accreditation group. After adjusting for potential covariates, CPC accreditation was associated with substantially reduced risks of in-hospital mortality (adjusted *OR* 0.644, 95% CI 0.599–0.693) and misdiagnosis (adjusted *OR* 0.554, 95% CI 0.493–0.624), along with an increase in the proportion of patients undergoing Stanford type A AAD surgery (adjusted *OR* 1.973, 95% CI 1.797–2.165). Following CPC accreditation, there were significant reductions in in-hospital mortality across various regions, particularly in Western regions (from 21.5 to 14.1%). Moreover, CPC accreditation demonstrated a more pronounced impact on in-hospital mortality in non-provincial cities compared to provincial cities (adjusted *OR* 0.607 vs. 0.713).

**Conclusion:**

CPC accreditation is correlated with improved management and in-hospital outcomes for patients with AAD.

**Supplementary Information:**

The online version contains supplementary material available at 10.1186/s40779-024-00565-0.

## Background

Acute aortic dissection (AAD) represents a critical cardiovascular emergency with high mortality rates [1–4] attributed to its rapid progression and potential for severe complications such as aortic rupture and cardiac tamponade [5–7]. Without prompt surgical intervention, acute Stanford type A AAD often leads to fatal outcomes [7, 8]. Therefore, accurate diagnosis and effective management of patients with AAD are essential for improving their in-hospital outcomes [9].

The National Chest Pain Center Program (NCPCP) is a pioneering, nationwide, hospital-based program designed to comprehensively and continuously enhance the quality of care for patients with acute chest pain in China. AAD presents primarily as chest pain and often requires differentiation from acute coronary syndrome (ACS) and acute pulmonary embolism (APE) [10, 11]. Despite AAD having a lower incidence than ACS, its in-hospital mortality rate of AAD is significantly higher [12–14]. Non-standardized procedures or misdiagnosis may result in catastrophic hemorrhage or exacerbation of AAD, particularly when thrombolytic drugs are used inappropriately [15, 16]. Therefore, some countries have implemented accreditation programs for chest pain centers (CPCs) to improve the quality of medical care for patients with chest pain. Previous studies conducted in both developed and developing countries have shown that CPC accreditation has effectively improved outcomes for ACS [17–20]. Although CPC accreditation imposes more stringent diagnostic and treatment processes for AAD [21], there is currently no evidence demonstrating its impact on patient management and in-hospital outcomes.

To the best of our knowledge, this study represents the initial exploration into whether CPC accreditation can improve in-hospital outcomes for AAD patients. Additionally, it illustrates the influence of CPC accreditation on the in-hospital outcomes of AAD patients across different geographical regions. Given the high in-hospital mortality rates associated with current treatment strategies for AAD, this study implies that CPC accreditation could offer a promising management strategy to improve outcomes for AAD patients.

## Methods

### Study design

In this retrospective observational study, a longitudinal self-contrast comparison design was employed to assess the impact of CPC accreditation on AAD patients based on the NCPCP. The NCPCP, also referred to as the CPC Accreditation Program, stands as an extensive nationwide initiative based in hospitals aimed at continuous enhancement of quality care provision. Its primary objective is establishing a regional emergency care network dedicated to managing patients with acute chest pain (including ACS, AAD, and APE) [22]. Participating hospitals were required to improve the quality of acute chest pain care while submitting relevant data. Upon completion of a minimum of 6-month data submission period, each hospital will undergo thorough online and on-site evaluations conducted by 3 experts from the NCPCP headquarters. The assessment covers various performance indicators, encompassing 5 accreditation standards. Hospitals meeting these criteria will be granted formal CPC accreditation. Those falling short are encouraged to refine their practices before reapplying for assessment. The accreditation criteria for NCPCP serve as a mechanism to enhance quality delineated across 5 dimensions. (1) Facility conditions, including the organization of CPCs and professional personnel. (2) Diagnosis and treatment process, including early and rapid screening of patients with acute chest pain and diagnosis and treatment of acute aortic dissection. (3) Integration of pre-hospital and hospital care. CPCs should establish a close cooperation mechanism with the pre-hospital emergency system. (4) Training and education, including training within the hospital and for other hospitals as well as community healthcare institutions in the region. (5) Capacity for continuous quality improvement. Hospitals should formulate plans and measures to promote process and quality improvement. The NCPCP headquarters provides training to assist in identifying and resolving any existing issues or obstacles.

The accreditation criteria for NCPCP for the diagnosis and management of AAD are as follows. (1) For patients with a high clinical suspicion of aortic dissection, an “enhanced CT scan” should be performed within 30 min from notification to the start of scanning. (2) In cases of suspected type A dissection, cardiac ultrasound examination should commence within 30 min. (3) An early emergency treatment plan for aortic dissection must be developed. Upon confirmed diagnosis and in the absence of contraindications, a treatment plan focusing on β-blockers and intravenous medications for blood pressure reduction and pain relief should be promptly implemented to mitigate the risk of aortic dissection rupture and expedite subsequent treatment. (4) A diagnostic and treatment flowchart for different types of aortic dissection needs to be established. If the hospital is equipped for surgical treatment of AAD, it is imperative to establish a multidisciplinary cooperation mechanism to ensure that patients receive appropriate treatment within the specified time frame outlined by professional guidelines. If the hospital lacks capacity for surgical treatment of AAD, it should establish referral relationships with hospitals possessing such capabilities to facilitate prompt transferal of unstable patients to facilities capable of providing optimal care. (5) Emergency physicians need to possess comprehensive knowledge regarding the clinical manifestations, diagnostic methods, and available treatments for AAD.

Ethics approvals for this study were obtained from the Institutional Review Boards of the Peking University Committee (2020-242). Informed consent was obtained from registered hospitals to collect data for research purposes.

### Study population

**Fig. 1:**
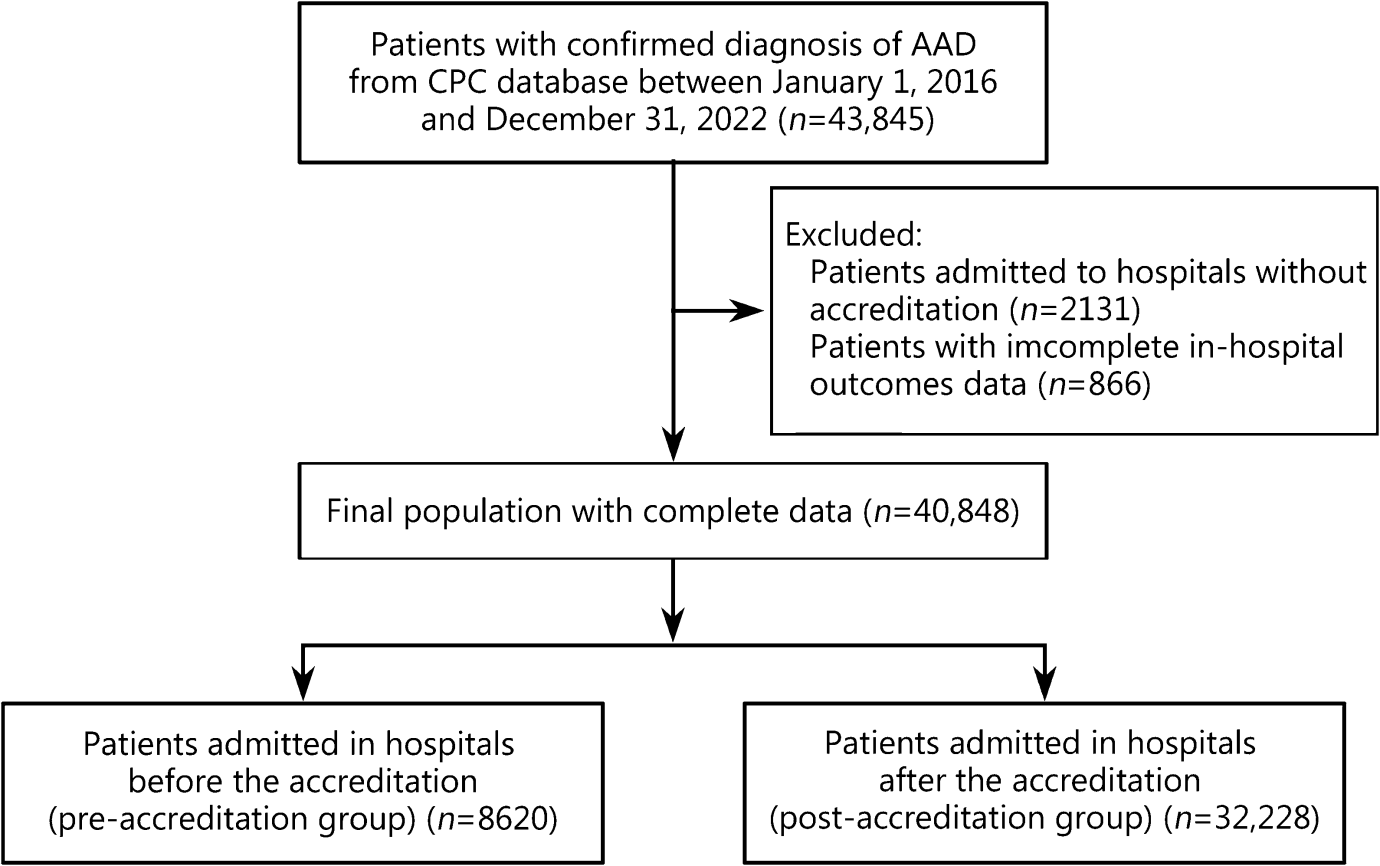
Flow diagram of selection of the study population. AAD acute aortic dissection, CPC chest pain center

From January 1, 2016 to December 31, 2022 (with data updated as of September 30, 2023), a total of 43,845 patients with a confirmed diagnosis of AAD were included for analysis from the CPC database. Exclusions comprised 2131 patients admitted to hospitals without accreditation and 866 patients with incomplete in-hospital outcome data. Finally, the dataset comprised 40,848 patients from 1671 accredited hospitals (Fig. 1; Additional file 1: Table S1). Consistent with prior research [23], patients admitted to hospitals before the corresponding accreditation date were categorized into the pre-accreditation group, while those admitted after the corresponding accreditation date were classified into the post-accreditation group.

To further examine the impact of the accreditation process status, the pre-accreditation group can be divided into two sub-groups: before accreditation and ongoing accreditation. Patients admitted to hospitals 6 months or more before the accreditation date are classified as ‘before accreditation’, while patients admitted within 6 months before the accreditation date are classified as ‘ongoing accreditation’, and those admitted after the accreditation date are classified as ‘post-accreditation’. The association between surgery and in-hospital mortality is statistically significant among patients diagnosed with Stanford type A AAD. To validate the findings, all Stanford type A AAD patients were categorized into surgical and non-surgical groups, which were subsequently subdivided into pre- and post-accreditation groups. Regional disparities have a notable impact on hospital outcomes for AAD. By categorizing hospitals according to their locations within Eastern, Central, and Western regions or based on their provincial capital/non-provincial capital status as per geographical location and previous research [24], we conducted further analysis to assess the influence of CPC accreditation across diverse regions.

### Data collection

The data for this study were obtained from the Chinese Cardiovascular Association Database-CPC. Participating hospitals, such as Zhongshan Hospital, and Fudan University, submitted data on patients with AAD through a centralized web-based registry system. AAD patients were identified using discharge diagnosis codes based on the 10th revision of the International Statistical Classification of Diseases (ICD-10).

### Outcome indicators

The primary outcome was in-hospital mortality. The secondary outcome included misdiagnosis and Stanford type A AAD surgery. In-hospital mortality was defined as death occurring during hospitalization. Misdiagnosis was characterized by a disparity between the initial diagnosis upon admission and the confirmed diagnosis. Stanford type A AAD surgery refers to surgical procedures performed on patients with Stanford type A AAD during hospitalization.

### Covariates

Patient demographic variables, onset symptoms, comorbidities, Stanford type A AAD, hospital location, and admission time (year) were recorded in the database. Patient demographic data encompassed age, sex (male or female), heart rate, and systolic blood pressure. Onset symptoms comprised dyspnea, sympathetic symptoms, abdominal pain, pain in the back or shoulders, toothache, and persistent chest pain.

### Statistical analysis

According to the Kolmogorov–Smirnov test, none of the continuous variables conform to a normal distribution. Therefore, the continuous variables were presented as median (*Q*_1_, *Q*_3_), while categorical variables were presented as *n* (%). Wilcoxon test was used for those exhibiting non-normal distribution. Chi‐squared tests were utilized to compare categorical variables.

We adopted a self-contrasted design between the pre-accreditation and post-accreditation groups to evaluate the management and in-hospital clinical outcomes in these hospitals before and after CPC accreditation. Mixed-effect models, including logistic regression models for in-hospital mortality, misdiagnosis, and Stanford type A AAD surgery, were applied. In the case of in-hospital mortality, adjustments were made for sex, age, heart rate, blood pressure, comorbidities (malignant arrhythmia, heart failure, syncope), type of aortic dissection, hospital location, and admission time (or not). For misdiagnosis, adjustments included sex, age, heart rate, blood pressure, symptoms (dyspnea, sympathetic symptoms, abdominal pain, shoulder and back pain, toothache, persistent chest pain), comorbidities (malignant arrhythmia, heart failure, syncope), hospital location, and admission time (or not). For Stanford type A AAD surgery, adjustments involved sex, age, heart rate, blood pressure, comorbidities (malignant arrhythmia, heart failure, syncope), hospital location, and admission time (or not). Multivariate logistic regression models were employed to explore the impact of CPC in the surgical or non-surgical groups among patients with Stanford type A AAD. Sensitivity analysis was performed by grouping patients based on age (≥ 65 years), gender, and hospital type. A two-tailed *P* < 0.05 was considered statistically significant in all analyses. All statistical analyses were performed using SPSS software (version 27).

## Results

### Characteristics of patients in pre-accreditation group and post-accreditation group

In this study, a total of 40,848 patients were enrolled from 1671 hospitals. The cohort included 8620 patients in the pre-accreditation group and 32,228 patients in the post-accreditation group (Fig. 1). Baseline characteristics are presented in Table 1. Compared with the pre-accreditation group, the post-accreditation group exhibited younger ages (57 vs. 59, *P* < 0.001), a higher proportion of males (77.2% vs. 75.0%, *P* < 0.001), and a higher incidence of Stanford type A AAD (38.4% vs. 32.5%, *P* < 0.001). Patients in the post-accreditation group more frequently presented with syncope as a concurrent complication (1.1% *vs.* 0.7%, *P* = 0.002), whereas occurrences of heart failure were less common (0.2% *vs.* 0.4%, *P* < 0.001).

**Table 1:** Baseline Characteristics and in-hospital outcomes of patients in pre-accreditation group and post-accreditation group

Characteristics	Pre-accreditation (*n* = 8620)	Post-accreditation (*n* = 32,228)	*P*-value
Age [year, median (*Q*_1_, *Q*_3_)]	59 (49, 70)	57 (48, 67)	< 0.001
Sex [*n* (%)]			
Male	6463 (75.0)	24,883 (77.2)	< 0.001
Female	2157 (25.0)	7345 (22.8)	< 0.001
Admission time [year, *n* (%)]			< 0.001
2016	592 (6.9)	719 (2.2)	
2017	1398 (16.2)	1576 (4.9)	
2018	2027 (23.5)	2823 (8.8)	
2019	2089 (24.2)	4299 (13.3)	
2020	1545 (17.9)	6373 (19.8)	
2021	748 (8.7)	7620 (23.6)	
2022	221 (2.6)	8818 (27.4)	
Heart rate [bpm, median (*Q*_1_, *Q*_3_)]	77 (66, 88)	77 (67, 88)	0.383
SBP [mmHg, median (*Q*_1_, *Q*_3_)]	152 (128, 179)	150 (128, 175)	0.001
Symptom [*n* (%)]			
Dyspnea	301 (3.5)	1230 (3.8)	0.159
Sympathetic symptoms	369 (4.3)	2400 (7.5)	< 0.001
Abdominal pain	1011 (11.7)	3215 (10.0)	< 0.001
Pain in the back or shoulders	509 (5.9)	4544 (14.1)	< 0.001
Toothache	68 (0.8)	419 (1.3)	< 0.001
Persistent chest pain	5644 (65.5)	19,859 (61.6)	< 0.001
Complication [*n* (%)]			
Malignant arrhythmia	12 (0.1)	28 (0.1)	0.168
Heart failure	34 (0.4)	55 (0.2)	< 0.001
Syncope	60 (0.7)	345 (1.1)	0.002
Stanford type A aortic dissection [*n* (%)]	2803 (32.5)	12,361 (38.4)	< 0.001
Hospital location: provincial capital [*n* (%)]	2350 (27.3)	11,509 (35.7)	< 0.001
In-hospital outcomes [*n* (%)]			
In-hospital mortality	1408 (16.3)	3909 (12.1)	< 0.001
Type A AAD	856/2803 (30.5)	2661/12,361 (21.5)	< 0.001
Type B AAD	552/5817 (9.5)	1248/19,867 (6.3)	< 0.001
Misdiagnosis	461 (5.4)	934 (2.9)	< 0.001
ACS	327 (3.8)	686 (2.1)	
STEMI	90 (1.0)	239 (0.7)	
NSTEMI	71 (0.8)	152 (0.5)	
UA	166 (1.9)	295 (0.9)	
Pulmonary embolism	16 (0.2)	79 (0.3)	
Other cardiogenic chest pain	37 (0.4)	96 (0.3)	
Non-cardiac chest pain	17 (0.2)	21 (0.1)	
Unknown etiology	64 (0.7)	52 (0.2)	
Standford type A AAD surgery	1179 (42.1)	7554 (61.1)	< 0.001

Data are expressed as median (*Q*_1_, *Q*_3_) or [*n* (%)]. *SBP* systolic blood pressure, *ACS* acute coronary syndrome, *STEMI* ST-segment elevation myocardial infarction, *NSTEMI* non-ST-segment elevation myocardial infarction, *UA* unstable angina, *AAD* acute aortic dissection

### CPC accreditation improves the hospital outcomes and management of patients with AAD

The in-hospital mortality rate was significantly lower in post-accreditation group compared with pre-accreditation group (12.1% vs. 16.3%, *P* < 0.001, Table 1). Specifically, for type A AAD, the in-hospital mortality rate decreased from 30.5 to 21.5% after accreditation, while for type B AAD, it decreased from 9.5 to 6.3% (Table 1). Univariate logistic regression analysis further demonstrated a significant reduction in in-hospital mortality associated with accreditation (*OR* 0.707, 95% CI 0.662–0.755, *P* < 0.001, Table 2). After adjusting for potential covariates, CPC accreditation was linked to a notable decrease of up to 36% in in-hospital mortality (adjusted *OR* 0.644, 95% CI 0.599–0.693, *P* < 0.001, Table 2; Additional file 1: Table S2).

**Table 2:** Univariate and multivariate logistic regression analysis for the association between in-hospital outcomes and CPC accreditation

Variable	Univariate analysis		Multivariate analysis without admission time		Multivariate analysis with admission time	
	Post-accreditation [*OR* (95% CI)]	*P*-value	Post-accreditation [*OR* (95% CI)]	*P*-value	Post-accreditation [*OR* (95% CI)]	*P*-value
In-hospital mortality	0.707 (0.662–0.755)	< 0.001	0.644^a^ (0.599–0.693)	< 0.001	0.727^d^ (0.671–0.789)	< 0.001
Misdiagnosis	0.528 (0.471–0.592)	< 0.001	0.554^b^ (0.493–0.624)	< 0.001	0.604^e^ (0.531–0.689)	< 0.001
Standford type A AAD surgery	2.165 (1.992–2.353)	< 0.001	1.973^c^ (1.797–2.165)	< 0.001	1.461^f^ (1.319–1.617)	< 0.001

^a^Adjusted for sex, age, heart rate, systolic blood pressure, comorbidities (malignant arrhythmia, heart failure, syncope), hospital location, and Stanford type A AAD

^b^Adjusted for sex, age, heart rate, systolic blood pressure, symptom (dyspnea, sympathetic symptoms, abdominal pain, pain in back or shoulders, toothache, persistent chest pain), comorbidities (malignant arrhythmia, heart failure, syncope) and hospital location

^c^Adjusted for sex, age, heart rate, systolic blood pressure, comorbidities (malignant arrhythmia, heart failure, syncope), and hospital location

^d^Adjusted for admission time (year), sex, age, heart rate, systolic blood pressure, comorbidities (malignant arrhythmia, heart failure, syncope), hospital location, and Stanford type A AAD

^e^Adjusted for admission time (year), sex, age, heart rate, systolic blood pressure, symptom (dyspnea, sympathetic symptoms, abdominal pain, pain in back or shoulders, toothache, persistent chest pain), comorbidities (malignant arrhythmia, heart failure, syncope) and hospital location

^f^Adjusted for admission time (year), sex, age, heart rate, systolic blood pressure, comorbidities (malignant arrhythmia, heart failure, syncope), and hospital location. *OR* odds ratio, *CI* confidence interval, *AAD* acute aortic dissection, *CPC* chest pain center

The misdiagnosis rate of AAD was significantly lower in the post-accreditation group compared with the pre-accreditation group (2.9% vs. 5.4%, *P* < 0.001, Table 1). Further analysis of the misdiagnosis revealed that over 70% of misdiagnosed patients were initially diagnosed with ACS upon admission (Table 1). Univariate analysis showed that CPC accreditation was associated with a lower misdiagnosis (*OR* 0.528, 95% CI 0.471–0.592, *P* < 0.001). After adjusting for possible confounding variables, particularly symptoms, CPC accreditation was associated with a significantly decreased risk of misdiagnosis (adjusted *OR* 0.554, 95% CI 0.493–0.624, *P* < 0.001, Table 2; Additional file 1: Table S3).

Stanford type A AAD surgery was used as a metric to assess the management of patients with AAD. The post-accreditation group exhibited a significantly higher Stanford type A AAD surgical rate than the pre-accreditation group (61.1% vs. 42.1%, *P* < 0.001, Table 1). Both univariate and multivariate logistic regression analyses indicated a positive correlation between CPC accreditation and Stanford type A AAD surgery (*OR* 2.165, 95% CI 1.992–2.353, *P* < 0.001; adjusted *OR* 1.973, 95% CI 1.797–2.165, *P* < 0.001, Table 2; Additional file 1: Table S4).

The mortality rate of AAD has decreased over time due to improved awareness of the disease and advancements in surgical techniques. Admission time may play a significant role in patient outcomes. Even after adjusting for admission time, CPC accreditation remains associated with superior in-hospital management and outcomes for AAD patients (Table 2; Additional file 1: Tables S5–7).

Given that surgical intervention is the preferred treatment for type A AAD and has been shown to reduce in-hospital mortality, we conducted a comparative analysis of baseline data between patients who underwent surgery and those who did not for type A AAD (Additional file 1: Table S8). Patients who underwent surgery were found to be younger and more frequently admitted to provincial capital hospitals (Additional file 1: Table S8). Even after adjusting for surgery and other potential influencing factors, CPC accreditation remained significantly associated with a reduction in in-hospital mortality among type A AAD patients (Additional file 1: Table S9). We also compared the baseline data and outcomes between pre-accreditation and post-accreditation periods for both surgical and non-surgical patients (Additional file 1: Table S10). In both the surgical and non-surgical groups, there was a decrease in in-hospital mortality among type A AAD patients following CPC accreditation. After adjusting for confounders, CPC accreditation consistently demonstrated an association with reduced in-hospital mortality in both the surgical and non-surgical groups (Additional file 1: Table S11).

### CPC accreditation status affected patients with AAD

Based on the accreditation process status, the pre-accreditation group can be divided into two sub-groups: before accreditation and ongoing accreditation. A comprehensive analysis was conducted to assess the impact of CPC accreditation status (before accreditation, ongoing accreditation, and post-accreditation) on various factors such as in-hospital mortality, misdiagnosis, and Stanford type A AAD surgery while controling for potential confounding variables (Table 3). When compared with before accreditation group, ongoing accreditation group demonstrated a modest decrease in suspected misdiagnosis (adjusted *OR* 0.810, 95% CI 0.664–0.988, *P* = 0.038), but no significant change was observed in predicted in-hospital mortality (adjusted *OR* 1.014, 95% CI 0.892–1.152, *P* = 0.832) or Stanford type A AAD surgery (adjusted *OR* 0.940, 95% CI 0.792–1.115, *P* = 0.478). After completing CPC accreditation, the post-accreditation group exhibited significant improvements in in-hospital management and prognosis. Specifically, there was a decrease in the risk of in-hospital mortality (adjusted *OR* 0.648, 95% CI 0.592–0.710, *P* < 0.001) and misdiagnosis (adjusted *OR* 0.510, 95% CI 0.443–0.586, *P* < 0.001), as well as a notable increase in the surgery for Stanford type A AAD (adjusted *OR* 1.923, 95% CI 1.711–2.161, *P* < 0.001) (Table 3).

**Table 3:** Univariate and multivariate logistic regression analysis for the association between in-hospital outcomes and CPC accreditation status

Variable		Before accreditation [*OR* (95% CI)]	Ongoing accreditation [*OR* (95% CI)]	*P-*value	Post-accreditation [*OR* (95% CI)]	*P-*value
In-hospital mortality	Unadjusted	Ref	1.027 (0.914–1.152)	0.657	0.715 (0.658–0.776)	< 0.001
	Adjusted	Ref	1.014^a^ (0.892–1.152)	0.832	0.648^a^ (0.592–0.710)	< 0.001
Misdiagnosis	Unadjusted	Ref	0.839 (0.692–1.018)	0.076	0.492 (0.430–0.564)	< 0.001
	Adjusted	Ref	0.810^b^ (0.664–0.988)	0.038	0.510^b^ (0.443–0.586)	< 0.001
Standford type A AAD surgery	Unadjusted	Ref	0.891 (0.765–1.038)	0.140	2.064 (1.860–2.291)	< 0.001
	Adjusted	Ref	0.940^c^ (0.792–1.115)	0.478	1.923^c^ (1.711–2.161)	< 0.001

^a^Adjusted for sex, age, heart rate, systolic blood pressure, comorbidities, hospital location, and Stanford type A AAD

^b^Adjusted for sex, age, heart rate, systolic blood pressure, symptom (dyspnea, sympathetic symptoms, abdominal pain, pain in back or shoulders, toothache, persistent chest pain), comorbidities (malignant arrhythmia, heart failure, syncope) and hospital location

^c^Adjusted for sex, age, heart rate, systolic blood pressure, comorbidities, and hospital location

*CPC* chest pain center, *OR* odds ratio, *CI* confidence interval, *Ref* reference, *AAD* acute aortic dissection

### CPC accreditation affected patients with AAD in different regions

After stratifying hospitals by Eastern, Central, and Western regions or by segmenting them into provincial capital/non-provincial capital status based on geographical location and previous research [24], we further investigated how CPC accreditation impacts improvements for AAD patients across various regions. Across different areas, CPC accreditation consistently demonstrates effective reductions in in-hospital mortality rate, and misdiagnosis rate, while increasing type A AAD surgical intervention rate (Table 4). Notably, AAD patients from the Western region exhibited higher in-hospital mortality rates compared with their counterparts from the Eastern and Central regions. Following CPC accreditation implementation, a substantial reduction in in-hospital mortality was observed among Western region’s patient outcomes (from 21.5 to 14.1%) (Table 4). Additionally, despite initially having lower rates of type A AAD surgery than other regions before accreditation; there was a significant increase post-accreditation in the Western (from 30.5 to 57.7%).

**Table 4:** In-hospital outcomes of patients in the pre-accreditation group and post-accreditation group among different hospital locations [*n* (%)]

Hospital location	Group	In-hospital mortality	Misdiagnosis	Standford type A AAD surgery
Regions				
Eastern	Pre-accreditation (*n* = 3408)	567 (16.6)	195 (5.7)	453 (43.1)
	Post-accreditation (*n* = 14,145)	1661 (11.7)	398/ (2.8)	3441 (62.1)
	*P*-value	< 0.001	< 0.001	< 0.001
Central	Pre-accreditation (*n* = 3263)	422 (12.9)	150 (4.6)	514 (48.6)
	Post-accreditation (*n* = 10,304)	1155 (11.2)	293 (2.8)	2459 (62.4)
	*P*-value	0.002	< 0.001	< 0.001
Western	Pre-accreditation (*n* = 1949)	419 (21.5)	116 (6.0)	212 (30.5)
	Post-accreditation (*n* = 7779)	1093 (14.1)	243 (3.1)	1654 (57.7)
	*P*-value	< 0.001	< 0.001	< 0.001
Provincial capital/non-provincial capital				
Non-provincial capital	Pre-accreditation (*n* = 6270)	1091 (17.4)	368 (5.9)	586 (30.6)
	Post-accreditation (*n* = 20,719)	2668 (12.9)	698 (3.4)	3514 (49.5)
	*P*-value	< 0.001	< 0.001	< 0.001
Provincial capital	Pre-accreditation (*n* = 2350)	317 (13.5)	93 (4.0)	593 (66.8)
	Post-accreditation (*n* = 11,509)	1241 (10.8)	236 (2.1)	4040 (76.7)
	*P*-value	< 0.001	< 0.001	< 0.001

*AAD* acute aortic dissection

Compared to provincial capital cities, patients with AAD in non-provincial capital cities exhibited higher mortality rates, misdiagnosis rates and lower A type AAD surgery rates (Table 4). Following the CPC accreditation of the hospital, the improvement was more significant for AAD patients in non-provincial cities.

### Sensitivity analysis

**Fig. 2:**
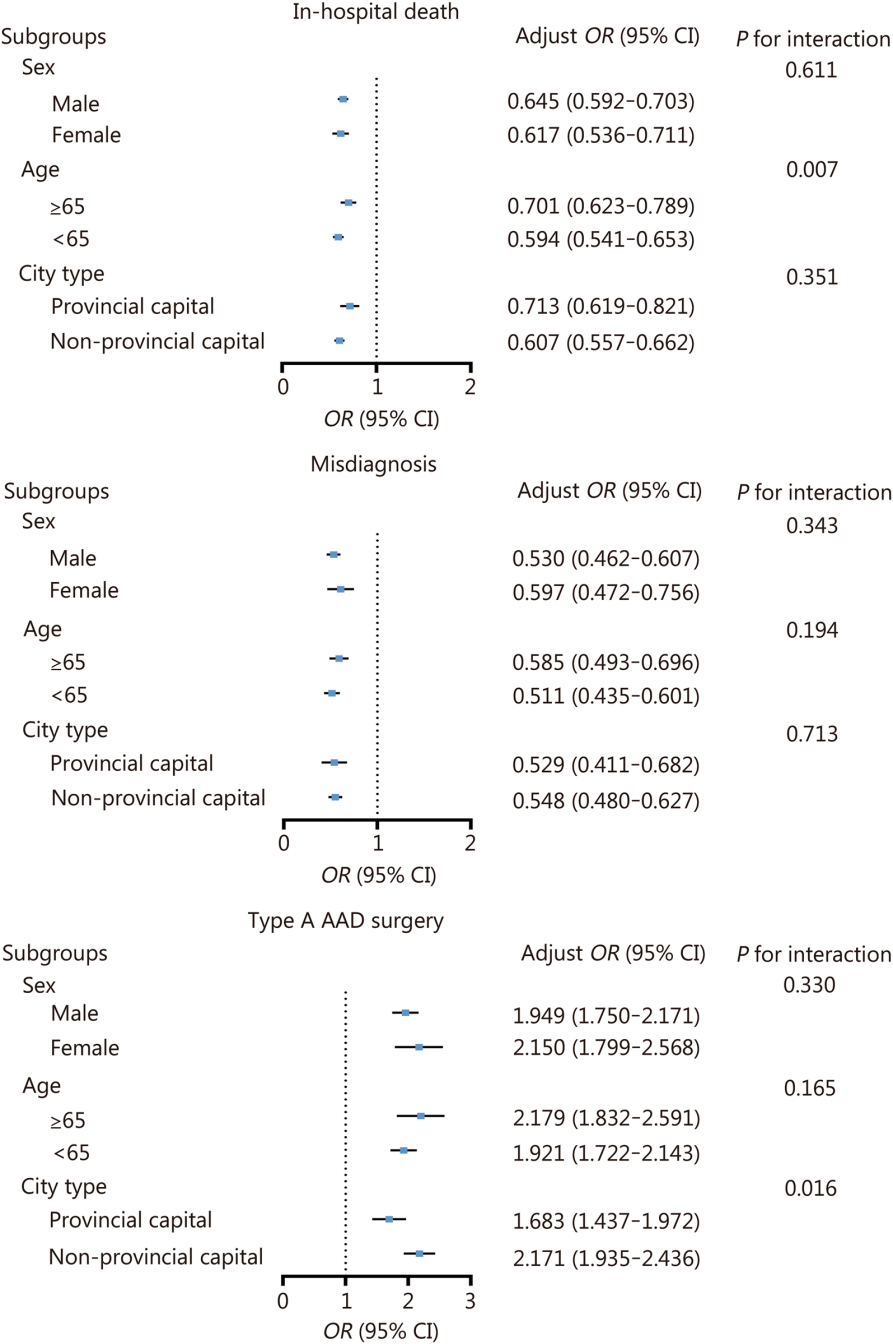
Sensitive analyses for the association between in-hospital outcomes and CPC accreditation. *OR* odds ratio, CI confidence interval, AAD acute aortic dissection, CPC chest pain center

To further validate the robustness of the findings presented in Table 2 against potential confounding factors, sensitive analyses were conducted based on age (≥ 65 years), sex, and hospital type. All analyses adjusted for the model utilized in Table 2, except for the stratified variable. The results from all analyses consistently demonstrated a positive association between CPC accreditation and improvements in AAD management and outcomes (Fig. 2). Notably, a significant interaction effect was observed between city type and CPC accreditation, showing a correlation with type A AAD surgery (*P* = 0.016). Specifically, CPC accreditation led to a substantial increase in the type A AAD surgical rate among non-provincial capital hospitals (adjusted *OR* 2.171, 95% CI 1.935–2.436), surpassing that seen in provincial capital hospitals (adjusted *OR* 1.683, 95% CI 1.437–1.972, Fig. 2). Moreover, it was found that CPC accreditation has a more pronounced impact on in-hospital mortality in non-provincial cities compared with provincial cities (adjusted *OR*: 0.607 vs. 0.713).

## Discussion

To the best of our knowledge, this study represents a comprehensive investigation into the impact of CPC accreditation on the management and clinical outcomes of patients with AAD. Our findings are as follows. (1) CPC accreditation demonstrates a significant reduction in in-hospital mortality among patients. (2) It markedly enhances the diagnostic accuracy of AAD and increases surgical intervention rates for Stanford type A AAD. (3) After CPC accreditation, improvements in the management and in-hospital outcomes of AAD patients were more pronounced in non-provincial capital areas compared with those in provincial capital areas. Taken together, these results suggest that CPC accreditation effectively improves both the in-hospital management and outcomes for AAD patients, providing a promising strategy for enhancing patient care.

CPC accreditation has been demonstrated to effectively improve standard medical care procedures and services in countries where it was implemented early [18]. Research conducted in China has established a strong correlation between CPC accreditation and improvements in hospitalized patient outcomes related to ACS [23]. The positive effects resulting from CPC accreditation regarding ACS have also been well-documented [17–20]. In addition to ACS being a leading cause of acute chest pain; however, its influence on managing and clinically impacting individuals suffering from AAD remains uncertain. Numerous factors contribute to predicting prognoses among those diagnosed with AAD, including their initial health status at presentation, categorization based on AAD, immediate interventions undertaken, and as well as hospital’s healthcare capabilities. Adjustments were made for potential confounders using multivariate regression analysis revealing increased age along with multiple comorbidities or type A AAD posed elevated risks associated with mortality, which is consistent with previous studies [25, 26]. Moreover, hospitals located in provincial capital cities or those with CPC accreditation may contribute to a reduction in the mortality risk of AAD, indicating the positive impact of higher hospital management quality on the prognosis of AAD. Our study illustrates that CPC accreditation leads to a significant improvement in the in-hospital outcomes of patients with AAD. After accounting for confounding variables, we have found that CPC accreditation is closely linked to a decrease in the in-hospital mortality rates of these patients. Given the current lack of effective medications specifically targeting AAD, surgery remains the primary treatment option upon admission [27]. This highlights the importance of prioritizing CPC accreditation to improve the in-hospital outcomes of patients with AAD.

Our research indicates that CPC accreditation improves the diagnostic accuracy of AAD, potentially leading to enhanced patient outcomes. Acute chest pain resulting from serious cardiovascular conditions such as ACS, AAD, and APE, is a common clinical presentation in emergency care settings. Accurate diagnosis is crucial for avoiding unnecessary treatments for non-target diseases, thereby minimizing harm to patients and conserving valuable healthcare resources [28]. Misdiagnosing AAD as ACS or APE can lead to severe consequences, such as catastrophic bleeding or exacerbation of AAD [15, 29], particularly if thrombolytic or anti-platelet medications are incorrectly administered [16]. Reducing the rate of AAD misdiagnosis is essential for proper patient management and treatment with the ultimate goal of improving their outcomes. The main reason for misdiagnosing patients with AAD, ACS, or APE lies in their similar symptoms’ presentations and the lack of diagnostic tools to differentiate these diseases. Electrocardiograms and chest X-rays have limited sensitivity and specificity, and definitive confirmatory techniques, such as computed tomography scans and magnetic resonance imaging, are often restricted or unavailable in emergency departments [9]. Therefore, the initial diagnosis of AAD heavily depends on the evaluation of the patient’s symptoms upon admission. The reduction in the misdiagnosis rate associated with CPC accreditation may be due to educational programs that enhance understanding and awareness of the acute state of AAD. Although CPC accreditation may not guarantee a correct diagnosis in every case, it does promote accurate prioritization and management of patients based on their symptoms. In ACS, enhancing diagnostic accuracy through the emergency medical dispatch system has the potential to reduce pre-hospital delays and improve survival outcomes [30, 31]. Thus, we speculate that the reduction in misdiagnosis rate due to CPC accreditation is a key factor contributing to improvements in the surgical rates and prognosis of patients with AAD. Our study not only revealed that CPC accreditation effectively improves diagnostic accuracy of AAD, but also demonstrated that the misdiagnosis rate for AAD is approximately 10–20% in China. In reality, this percentage may be even higher as many AAD patients pass away before receiving an accurate diagnosis [32]. Furthermore, our findings have identified ACS as the primary condition commonly misdiagnosed, highlighting the necessity for further training to distinguish it from AAD.

The leading cause of in-hospital mortality among patients with AAD is Stanford type A AAD [27, 33]. Guidelines recommended surgery as the primary treatment option to improve their prognosis [7]. Our research indicates that CPC accreditation significantly increases the surgical rate for Stanford type A AAD. Therefore, we hypothesize that this rise in surgical rates following CPC accreditation may be attributed to the standardization of acute chest pain assessment processes, improvements in interdepartmental collaboration systems, and enhancements in medical staff education.

Hospitals undergo a continuous process consisting of 3 stages to achieve accredited status.: before accreditation, ongoing accreditation, and post-accreditation. When seeking accreditation, hospitals are required to submit their data to NCPCP headquarters’ website for at least 6 months and undergo training provided by NCPCP headquarters aimed at enhancing their in-hospital chest pain diagnostic processes. This period is considered to be the ongoing accreditation stage. Our analysis indicates that the post-accreditation status yields the most positive impact on AAD outcomes. However, Sun et al. [17] have reported that notable improvements in in-hospital mortality resulting from acute myocardial infarction occur during the ongoing accreditation rather than post-accreditation stage. We speculate that this variance may be related to the timing of CPC accreditation program implementation and specific disease types.

The regional disparities in the management and prognosis of patients with AAD are multifaceted [27, 34, 35]. Potential factors contributing to these variations may include the accessibility of medical resources, the standard of care provided by physicians, and the integration of the healthcare systems [27, 35–37]. Generally speaking, patients with AAD received suboptimal treatment and had poorer prognoses in hospitals located in Western regions and non-provincial capitals. This may be attributed to lower economic development and limited medical resources in these areas. Importantly, following the implementation of the CPC accreditation program, there was a marked improvement in the management and prognosis of AAD patients in the Western region and non-provincial capital areas, leading to a reduction in regional differences. The primary driver behind this improvement is that CPC accreditation requires standardized care for all AAD patients, effectively reducing regional differences while promoting treatment standardization and improving service consistency for AAD patients. Nevertheless, regional discrepancies still exist even after accreditation. In the future, heightened attention and measures are imperative to elevate the level of management and care in remote regions while striving to eliminate inequalities in treatment quality across different regions.

According to the data from the International Registry of Acute Aortic Dissection [14, 38], the overall in-hospital mortality rate of type A AAD has decreased from approximately 30 to 15% over the past few decades. Our findings indicate that following CPC accreditation, the in-hospital mortality rate of type A AAD decreased from 30 to 20%. When compared with the data from the International Registry of Acute Aortic Dissection, which primarily includes hospitals in Europe and North America, the mortality rate in our study seems to be higher, possibly due to the inclusion of more non-provincial hospitals. This also suggests that further improvements are needed for AAD treatment in China.

The improvements in the management and in-hospital outcomes of AAD following CPC accreditation can be attributed to several factors. (1) Accreditation has optimized in-hospital diagnostic and treatment processes, leading to more accurate identification of AAD in patients and reduced misdiagnosis rate. (2) CPC accreditation promotes closer collaboration between the emergency department and emergency medical services, resulting in decreased time for managing acute-phase blood pressure and heart rate. Additionally, the wider promotion of CPC accreditation may have contributed to increased awareness among healthcare providers and the public regarding proper AAD diagnostic and treatment practices.

This study possesses several limitations. Firstly, being a retrospective, observational investigation entails inherent design deficiencies. This inclusion of sole patients from hospitals applying for CPC accreditation precludes comparison between those engaged and unengaged with the program’s enhancement efforts. Future endeavors should consider incorporating non-participating hospitals or conducting randomized controlled trials for comprehensive exploration. Furthermore, our scrutiny exclusively centers on immediate hospital-based consequences without addressing the enduring effects of CPC accreditation, an area warranting further inquiry. Finally, since this study was conducted in China, its findings should be carefully generalized to other countries. It is worth exploring whether this model will work as well in other countries.

## Conclusions

Leveraging a comprehensive, nationwide registry dataset, CPC accreditation demonstrates an association with enhanced management of patients experiencing AAD during emergency admission and improved inpatient clinical outcomes. These include reduced in-hospital mortality rate, decreased misdiagnosis rate, and increased Stanford type A AAD surgical rate. This investigation indicates that CPC accreditation serves as an effective measure for augmenting the diagnosis and treatment of AAD, thereby leading to improved prognostic outcomes.

### Supplementary Information

**Additional file 1: Table S1** Distribution of chest pain centers involved in our analysis. **Table S2** Multivariate logistic regression analysis for the association between in-hospital mortality and CPC accreditation. **Table S3** Multivariate logistic regression analysis for the association between misdiagnosis and CPC accreditation. **Table S4** Multivariate logistic regression analysis for the association between Stanford type A AAD surgery and CPC accreditation. **Table S5** Multivariate logistic regression analysisfor the association between in-hospital mortality and CPC accreditation. **Table S6** Multivariate logistic regression analysisfor the association between misdiagnosis and CPC accreditation. **Table S7** Multivariate logistic regression analysisfor the association between Stanford type A AAD surgery and CPC accreditation. **Table S8** Baseline Characteristics of Stanford type A AAD patients in the surgery group and non-surgery group. **Table S9** Univariate and multivariate logistic regression analysis for the association between in-hospital death for Stanford type A AAD and CPC accreditation. **Table S10** Baseline Characteristics of Stanford type A AAD patients in the surgery group and non-surgery group before and after accreditation. **Table S11** Univariate and multivariate logistic regression analysis for the association between in-hospital death for Stanford type A AAD and CPC accreditation in the surgery/non-surgery group.

## Data Availability

Anonymized data will be accessible through a formal application process that will undergo review by the Data Management Committee of the Chinese Cardiovascular Association Database-Chest Pain Center.

## Abbreviations

NCPCP: The National Chest Pain Center Program
CPC: Chest pain center
ACS: Acute coronary syndrome
AAD: Acute aortic dissection
APE: Acute pulmonary embolism
SBP: Systolic blood pressure
STEMI: ST-segment elevation myocardial infarction
NSTEMI: Non-ST-segment elevation myocardial infarction
UA: Unstable angina

## References

[1] Murphy SL, Xu J, Kochanek KD, Arias E, Tejada-Vera B. Deaths: final data for 2018. Natl Vital Stat Rep. 2021;69(13):1–83. 33541516

[2] Wang SW, Huang YB, Huang JW, Chiu CC, Lai WT, Chen CY. Epidemiology, clinical features, and prescribing patterns of aortic aneurysm in Asian population from 2005 to 2011. Medicine (Baltimore). 2015;94(41):e1716. 26469911 10.1097/MD.0000000000001716 PMC4616784

[3] Bossone E, Corteville DC, Harris KM, Suzuki T, Fattori R, Hutchison S, et al. Stroke and outcomes in patients with acute type a aortic dissection. Circulation. 2013;128(11 Suppl 1):S175–9. 24030403 10.1161/CIRCULATIONAHA.112.000327

[4] Benjamin EJ, Blaha MJ, Chiuve SE, Cushman M, Das SR, Deo R, et al. Heart disease and stroke statistics-2017 update: a report from the American heart association. Circulation. 2017;135(10):e146-603. 28122885 10.1161/CIR.0000000000000485 PMC5408160

[5] Carrel T, Sundt TM 3rd, von Kodolitsch Y, Czerny M. Acute aortic dissection. Lancet. 2023;401(10378):773–88. 36640801 10.1016/S0140-6736(22)01970-5

[6] Guo MH, Appoo JJ, Saczkowski R, Smith HN, Ouzounian M, Gregory AJ, et al. Association of mortality and acute aortic events with ascending aortic aneurysm: a systematic review and meta-analysis. JAMA Netw Open. 2018;1(4):e181281. 30646119 10.1001/jamanetworkopen.2018.1281 PMC6324275

[7] Martin C, Martin G, Tim B, Victor A, Alessandro Della C, Edward PC, et al. EACTS/STS Guidelines for diagnosing and treating acute and chronic syndromes of the aortic organ. Eur J Cardio-Thoracic Surg. 2024;65:ezad426. 10.1093/ejcts/ezad426 38408364

[8] Eikelboom R, Katz N. Acute aortic dissection presenting as status epilepticus. CMAJ. 2020;192(11):E283–5. 32179537 10.1503/cmaj.190822 PMC7083549

[9] Olin JW, Fuster V. Acute aortic dissection: the need for rapid, accurate, and readily available diagnostic strategies. Arterioscler Thromb Vasc Biol. 2003;23(10):1721–3. 14555642 10.1161/01.ATV.0000093222.33222.D2

[10] Ohle R, Kareemi HK, Wells G, Perry JJ. Clinical examination for acute aortic dissection: a systematic review and meta-analysis. Acad Emerg Med. 2018;25(4):397–412. 29265487 10.1111/acem.13360

[11] Bazarian JJ, Bennett RS. Chest pain and aortic dissection. Ann Emerg Med. 1995;26(4):526–7. 7574139 10.1016/S0196-0644(95)70125-7

[12] Yin J, Liu F, Wang J, Yuan P, Wang S, Guo W. Aortic dissection: global epidemiology. Cardiol Plus. 2022;7:151–61. 10.1097/CP9.0000000000000028

[13] Shuduo Z, Yan Z, Xuejie D, Junxiong M, Na L, Hong S, et al. Regional variations in management and outcomes of patients with acute coronary syndrome in China: evidence from the National Chest Pain Center Program. Sci Bull. 2024;69:1302–12. 10.1016/j.scib.2024.03.010 38519397

[14] Evangelista A, Isselbacher EM, Bossone E, Gleason TG, Eusanio MD, Sechtem U, et al. Insights from the international registry of acute aortic dissection: a 20-year experience of collaborative clinical research. Circulation. 2018;137(17):1846–60. 29685932 10.1161/CIRCULATIONAHA.117.031264

[15] Blankenship JC, Almquist AK. Cardiovascular complications of thrombolytic therapy in patients with a mistaken diagnosis of acute myocardial infarction. J Am Coll Cardiol. 1989;14(6):1579–82. 2809020 10.1016/0735-1097(89)90402-6

[16] Wilcox RG, von der Lippe G, Olsson CG, Jensen G, Skene AM, Hampton JR. Trial of tissue plasminogen activator for mortality reduction in acute myocardial infarction: Anglo-Scandinavian Study of Early Thrombolysis (ASSET). Lancet. 1988;2(8610):525–30. 2900919 10.1016/S0140-6736(88)92656-6

[17] Sun P, Li J, Fang W, Su X, Yu B, Wang Y, et al. Effectiveness of chest pain centre accreditation on the management of acute coronary syndrome: a retrospective study using a national database. BMJ Qual Saf. 2021;30(11):867–75. 33443197 10.1136/bmjqs-2020-011491

[18] Ross MA, Amsterdam E, Peacock WF, Graff L, Fesmire F, Garvey JL, et al. Chest pain center accreditation is associated with better performance of centers for medicare and medicaid services core measures for acute myocardial infarction. Am J Cardiol. 2008;102(2):120–4. 18602506 10.1016/j.amjcard.2008.03.028

[19] Gibler WB, Runyon JP, Levy RC, Sayre MR, Kacich R, Hattemer CR, et al. A rapid diagnostic and treatment center for patients with chest pain in the emergency department. Ann Emerg Med. 1995;25(1):1–8. 7802357 10.1016/S0196-0644(95)70347-0

[20] Bahr RD. Chest pain centers: moving toward proactive acute coronary care. Int J Cardiol. 2000;72(2):101–10. 10646950 10.1016/S0167-5273(99)00160-6

[21] Bahr RD, Copeland C, Strong J. Chest pain centers–part 2: the strategy of the chest pain center. J Cardiovasc Manag. 2002;13(2):21–2. 11930818

[22] Dingcheng X, Yinzi J, Wei-Yi F, Xi S, Bo Y, Yan W, et al. The national chest pain centers program: monitoring and improving quality of care for patients with acute chest pain in China. Cardiol Plus. 2021;6:187–97. 10.4103/2470-7511.327239

[23] Fan F, Li Y, Zhang Y, Li J, Liu J, Hao Y, et al. Chest pain center accreditation is associated with improved in-hospital outcomes of acute myocardial infarction patients in China: Findings from the CCC-ACS project. J Am Heart Assoc. 2019;8(21):e013384. 31630594 10.1161/JAHA.119.013384 PMC6898834

[24] Liu Q, Guo Y. Regional differences of individual and allocation efficiencies of health resources in China. Front Public Health. 2023;11:1306148. 38179567 10.3389/fpubh.2023.1306148 PMC10764467

[25] Bossone E, Gorla R, LaBounty TM, Suzuki T, Gilon D, Strauss C, et al. Presenting systolic blood pressure and outcomes in patients with acute aortic dissection. J Am Coll Cardiol. 2018;71(13):1432–40. 29598863 10.1016/j.jacc.2018.01.064

[26] Tolenaar JL, Froehlich W, Jonker FH, Upchurch GR Jr, Rampoldi V, Tsai TT, et al. Predicting in-hospital mortality in acute type b aortic dissection: evidence from international registry of acute aortic dissection. Circulation. 2014;130(11 Suppl 1):S45-50. 25200055 10.1161/CIRCULATIONAHA.113.007117

[27] Erbel R, Aboyans V, Boileau C, Bossone E, Bartolomeo RD, Eggebrecht H, et al. 2014 ESC guidelines on the diagnosis and treatment of aortic diseases: Document covering acute and chronic aortic diseases of the thoracic and abdominal aorta of the adult. The task force for the diagnosis and treatment of aortic diseases of the European Society of Cardiology (ESC). Eur Heart J. 2014;35(41):2873–926. 25173340 10.1093/eurheartj/ehu281

[28] Hagan PG, Nienaber CA, Isselbacher EM, Bruckman D, Karavite DJ, Russman PL, et al. The international registry of acute aortic dissection (IRAD): new insights into an old disease. JAMA. 2000;283(7):897–903. 10685714 10.1001/jama.283.7.897

[29] Butler J, Davies AH, Westaby S. Streptokinase in acute aortic dissection. BMJ. 1990;300(6723):517–9. 2107933 10.1136/bmj.300.6723.517 PMC1662323

[30] Dong X, Ding F, Zhou S, Ma J, Li N, Maimaitiming M, et al. Optimizing an emergency medical dispatch system to improve prehospital diagnosis and treatment of acute coronary syndrome: nationwide retrospective study in China. J Med Internet Res. 2022;24(11):e36929. 36416876 10.2196/36929 PMC9730207

[31] Golden AP, Odoi A. Emergency medical services transport delays for suspected stroke and myocardial infarction patients. BMC Emerg Med. 2015;15:34. 26634914 10.1186/s12873-015-0060-3 PMC4668620

[32] Nienaber CA, Clough RE, Sakalihasan N, Suzuki T, Gibbs R, Mussa F, et al. Aortic dissection. Nat Rev Dis Primers. 2016;2:16053. 27440162 10.1038/nrdp.2016.53

[33] van Arsdell GS, David TE, Butany J. Autopsies in acute type A aortic dissection. Surgical implications. Circulation. 1998;98(19 Suppl):299–302 (**discussion II-4**). 9852918

[34] Higo Y, Sawayama Y, Takashima N, Harada A, Yano Y, Yamamoto T, et al. Epidemiology of acute aortic dissection in a general population of 1.4 million people in Japan - Shiga Stroke and heart attack registry. Circ J. 2023;87(9):1155–61. 37211402 10.1253/circj.CJ-22-0758

[35] Kanaoka K, Iwanaga Y, Nakai M, Sumita Y, Saito Y, Miyamoto Y. Temporal trends and regional variations in cardiovascular care in Japan, 2010–2019. Int Heart J. 2023;64(1):53–9. 36725073 10.1536/ihj.22-445

[36] Jacobs JW, Shih AW, Lombard FW, Bartoszko J, Mullane D, Cserti-Gadzewich C, et al. A multidisciplinary comparison of transfusion and perioperative support for high-risk cardiac surgery at three large academic centres in North America. Transfus Med. 2023;33(4):337–48. 37170673 10.1111/tme.12972

[37] Tanaka A, Hebert AM, Smith-Washington A, Hoffstaetter T, Goldenberg R, Vemulapalli S, et al. Knowledge gaps in surgical management for aortic dissection. Semin Vasc Surg. 2022;35(1):35–42. 35501039 10.1053/j.semvascsurg.2022.02.009

[38] Huckaby LV, Sultan I, Trimarchi S, Leshnower B, Chen EP, Brinster DR, et al. Sex-based aortic dissection outcomes from the international registry of acute aortic dissection. Ann Thoracic Surg. 2022;113:498–505. 10.1016/j.athoracsur.2021.03.100 PMC9015734 34090668

