# Supplementary material for: Effectiveness of chest pain center accreditation on the hospital outcome of acute aortic dissection: a nationwide study in China
Source: Mil Med Res. 2024 Aug 26;11:62. doi: 10.1186/s40779-024-00565-0 (PMC11346265; doi:10.1186/s40779-024-00565-0)
Supplement: Supplementary file 1 — Additional file 1: Table S1 Distribution of chest pain centers involved in our analysis. Table S2 Multivariate logistic regression analysis for the association between in-hospital mortality and CPC accreditation. Table S3 Multivariate logistic regression analysis for the association between misdiagnosis and CPC accreditation. Table S4 Multivariate logistic regression analysis for the association between Stanford type A AAD surgery and CPC accreditation. Table S5 Multivariate logistic regression analysisfor the association between in-hospital mortality and CPC accreditation. Table S6 Multivariate logistic regression analysisfor the association between misdiagnosis and CPC accreditation. Table S7 Multivariate logistic regression analysisfor the association between Stanford type A AAD surgery and CPC accreditation. Table S8 Baseline Characteristics of Stanford type A AAD patients in the surgery group and non-surgery group. Table S9 Univariate and multivariate logistic regression analysis for the association between in-hospital death for Stanford type A AAD and CPC accreditation. Table S10 Baseline Characteristics of Stanford type A AAD patients in the surgery group and non-surgery group before and after accreditation. Table S11 Univariate and multivariate logistic regression analysis for the association between in-hospital death for Stanford type A AAD and CPC accreditation in the surgery/non-surgery group. [file 40779_2024_565_MOESM1_ESM.pdf]

Table S1 Distribution of chest pain centers involved in our analysis

Region	Number of CPC hospitals
Eastern	
Beijing	12
Fujian	35
Guangdong	138
Hainan	12
Hebei	99
Jiangsu	66
Liaoning	23
Shandong	127
Shanghai	33
Tianjin	13
Zhejiang	67
Central	
Anhui	50
Heilongjiang	40
Henan	152
Hubei	90
Hunan	61
Jiangxi	58
Jilin	27
Shanxi	31
Western	
Gansu	53
Chongqing	24
Guangxi	57
Guizhou	49
Inner Mongolia	35
Ningxia	11
Qinghai	3
Shaanxi	63
Sichuan	83
Xinjiang	39
Yunnan	117
Xizang	3

CPC chest pain center

Table S2 Multivariate logistic regression analysis for the association between in-hospital mortality and CPC accreditation

**Variable**	***OR* (95% CI)**	***P*-value**
Age	1.016 (1.013 − 1.018)	< 0.001
Male sex	1.042 (0.968 − 1.122)	0.271
Heart rate	1.003 (1.001 − 1.005)	0.001
SBP	0.982 (0.981 − 0.983)	< 0.001
Malignant arrhythmia	3.357 (1.655 − 6.807)	0.001
Heart failure	2.001 (1.215 − 3.294)	0.006
Syncope	2.557 (2.052 − 3.187)	< 0.001
Stanford-A AAD	3.486 (3.262 − 3.726)	< 0.001
Provincial capital hospital	0.647 (0.604 − 0.694)	< 0.001
Post-accreditation	0.644 (0.599 − 0.693)	< 0.001

The model was adjusted for sex, age, heart rate, systolic blood pressure, comorbidities (malignant arrhythmia, heart failure, syncope), hospital location and Stanford type A AAD. *CPC* chest pain center, *OR* odds ratio, *CI* confidence interval, *SBP* systolic blood pressure, *AAD* acute aortic dissection

Table S3 Multivariate logistic regression analysis for the association between misdiagnosis and CPC accreditation

**Variable**	***OR* (95% CI)**	***P*-value**
Age	1.022 (1.018 − 1.026)	< 0.001
Male sex	1.097 (0.965 − 1.247)	0.157
Heart rate	1.000 (0.997 − 1.003)	0.835
SBP	0.992 (0.991 − 0.993)	< 0.001
Dyspnea	1.960 (1.563 − 2.459)	< 0.001
Sympathetic symptoms	1.409 (1.144 − 1.736)	0.001
Abdominal pain	0.357 (0.277 − 0.459)	< 0.001
Pain in the back or shoulders	0.661 (0.543 − 0.804)	< 0.001
Toothache	0.990 (0.633 − 1.547)	0.963
Persistent chest pain	0.747 (0.667 − 0.837)	< 0.001
Malignant arrhythmia	4.200 (1.803 − 9.785)	0.001
Heart failure	1.961 (1.017 − 3.780)	0.044
Syncope	1.329 (0.903 − 1.955)	0.149
Provincial capital hospital	0.615 (0.541 − 0.700)	< 0.001
Post-accreditation	0.554 (0.493 − 0.624)	< 0.001

The model was adjusted for sex, age, heart rate, systolic blood pressure, symptom (dyspnea, sympathetic symptoms, abdominal pain, pain in back or shoulders, toothache, persistent chest pain), comorbidities (malignant arrhythmia, heart failure, syncope) and hospital location. *CPC* chest pain center, *OR* odds ratio, *CI* confidence interval, *SBP* systolic blood pressure

Table S4 Multivariate logistic regression analysis for the association between Stanford type A AAD surgery and CPC accreditation

**Variable**	***OR* (95% CI)**	***P*-value**
Age	0.952 (0.950 − 0.955)	< 0.001
Male sex	1.029 (0.946 − 1.118)	0.507
Heart rate	0.998 (0.996 − 1.000)	0.084
SBP	0.998 (0.997 − 0.999)	< 0.001
Malignant arrhythmia	0.477 (0.150 − 1.515)	0.209
Heart failure	0.305 (0.146 − 0.637)	0.002
Syncope	0.834 (0.646 − 1.075)	0.161
Provincial capital city	3.341 (3.097 − 3.603)	< 0.001
Post-accreditation	1.973 (1.797 − 2.165)	< 0.001

The model was adjusted for sex, age, heart rate, systolic blood pressure, comorbidities (malignant arrhythmia, heart failure, syncope), and hospital location. *AAD* acute aortic dissection, *CPC* chest pain center, *OR* odds ratio, *CI* confidence interval, *SBP* systolic blood pressure

Table S5 Multivariate logistic regression analysis (with year) for the association between in-hospital mortality and CPC accreditation

**Variable**	***OR* (95% CI)**	***P*-value**
Age	1.016 (1.013 − 1.018)	< 0.001
Male sex	1.043 (0.969 − 1.122)	0.266
Heart rate	1.003 (1.001 − 1.005)	0.001
SBP	0.982 (0.981 − 0.983)	< 0.001
Malignant arrhythmia	3.386 (1.668 − 6.875)	0.001
Heart failure	2.015 (1.222 − 3.322)	0.006
Syncope	2.773 (2.222 − 3.460)	< 0.001
Stanford-A AAD	3.619 (3.383 − 3.871)	< 0.001
Provincial capital hospital	0.644 (0.600 − 0.690)	< 0.001
Post-accreditation	0.727 (0.671 − 0.789)	< 0.001
Admission time		
2016	Ref	
2017	1.112 (0.887 − 1.394)	0.359
2018	1.047 (0.846 − 1.296)	0.674
2019	1.044 (0.847 − 1.286)	0.688
2020	0.879 (0.714 − 1.081)	0.221
2021	0.853 (0.692 − 1.051)	0.136
2022	0.746 (0.605 − 0.920)	0.006

The model was adjusted for admission time (year), sex, age, heart rate, systolic blood pressure, comorbidities (malignant arrhythmia, heart failure, syncope), hospital location, and Stanford type A AAD. *CPC* chest pain center, *OR* odds ratio, *CI* confidence interval, *Ref* reference, *SBP* systolic blood pressure, *AAD* acute aortic dissection

Table S6 Multivariate logistic regression analysis (with year) for the association between misdiagnosis and CPC accreditation

**Variable**	***OR* (95% CI)**	***P*-value**
Age	1.022 (1.018 − 1.026)	< 0.001
Male sex	1.095 (0.963 − 1.245)	0.165
Heart rate	1.000 (0.997 − 1.003)	0.851
SBP	0.992 (0.991 − 0.993)	< 0.001
Dyspnea	1.964 (1.565 − 2.465)	< 0.001
Sympathetic symptoms	1.494 (1.207 − 1.848)	< 0.001
Abdominal pain	0.359 (0.279 − 0.462)	< 0.001
Pain in the back or shoulders	0.700 (0.573 − 0.856)	0.001
Toothache	1.029 (0.657 − 1.610)	0.901
Persistent chest pain	0.742 (0.662 − 0.832)	< 0.001
Malignant arrhythmia	4.432 (1.908 − 10.295)	0.001
Heart failure	1.968 (1.021 − 3.793)	0.043
Syncope	1.422 (0.964 − 2.097)	0.076
Provincial capital hospital	0.620 (0.544 − 0.705)	< 0.001
Post-accreditation	0.604 (0.531 − 0.689)	< 0.001
Admission time		
2016	Ref	
2017	1.560 (1.042 − 2.335)	0.031
2018	1.607 (1.092 − 2.365)	0.016
2019	1.310 (0.892 − 1.925)	0.168
2020	1.188 (0.808 − 1.747)	0.381
2021	1.231 (0.834 − 1.816)	0.295
2022	1.102 (0.743 − 1.633)	0.630

The model was adjusted for admission time (year), sex, age, heart rate, systolic blood pressure, symptom (dyspnea, sympathetic symptoms, abdominal pain, pain in back or shoulders, toothache, persistent chest pain), comorbidities (malignant arrhythmia, heart failure, syncope) and hospital location. *CPC* chest pain center, *OR* odds ratio, *CI* confidence interval, *Ref* reference, *SBP* systolic blood pressure

Table S7 Multivariate logistic regression analysis (with year) for the association between Stanford type A AAD surgery and CPC accreditation

**Variable**	***OR* (95% CI)**	***P*-value**
Age	0.951 (0.948 − 0.954)	< 0.001
Male sex	1.031 (0.948 − 1.121)	0.481
Heart rate	0.998 (0.996 − 1.000)	0.072
SBP	0.998 (0.996 − 0.999)	< 0.001
Malignant arrhythmia	0.438 (0.137 − 1.400)	0.164
Heart failure	0.309 (0.147 − 0.651)	0.002
Syncope	0.710 (0.549 − 0.918)	0.009
Provincial capital city	3.479 (3.222 − 3.757)	< 0.001
Post-accreditation	1.461 (1.319 − 1.671)	< 0.001
Admission time		
2016	Ref	
2017	1.594 (1.179 − 2.157)	0.002
2018	1.717 (1.298 − 2.271)	< 0.001
2019	1.692 (1.288 − 2.221)	< 0.001
2020	2.316 (1.809 − 3.183)	< 0.001
2021	2.596 (1.987 − 3.392)	< 0.001
2022	3.616 (2.765 − 4.730)	< 0.001

The model was adjusted for admission time (year), sex, age, heart rate, systolic blood pressure, comorbidities (malignant arrhythmia, heart failure, syncope), and hospital location. *AAD* acute aortic dissection, *CPC* chest pain center, *OR* odds ratio, *CI* confidence interval, *Ref* reference, *SBP* systolic blood pressure

Table S8 Baseline Characteristics of Stanford type A AAD patients in the surgery group and non-surgery group

**Characteristics**	**Surgical group (n = 8733)**	**Non-surgical group (n = 6431)**	***P*-value**
Age [year, median (Q_1, Q_3)]	53 (45, 62)	63 (52, 72)	< 0.001
Sex [n (%)]			
Male	6714 (76.9)	4389 (68.2)	< 0.001
Female	2019 (23.1)	2042 (31.8)	< 0.001
Heart rate [bpm, median (Q_1, Q_3)]	77 (66, 88)	75 (64, 88)	< 0.001
SBP [mmHg, median (Q_1, Q_3)]	138 (118, 160)	140 (116, 167)	0.014
Symptom [n (%)]			
Dyspnea	321(3.7)	312 (4.9)	< 0.001
Sympathetic symptoms	676 (7.7)	521(8.1)	0.416
Abdominal pain	622 (7.1)	639 (9.9)	< 0.001
Pain in the back or shoulders	1166 (13.4)	728 (11.3)	< 0.001
Toothache	124 (1.4)	124 (1.9)	0.015
Persistent chest pain	5700 (65.3)	4035 (62.7)	0.001
Complication [n (%)]			
Malignant arrhythmia	7 (0.1)	8 (0.1)	0.392
Heart failure	11 (0.1)	35 (0.5)	< 0.001
Syncope	150 (1.7)	152 (2.4)	0.005
Hospital location: provincial capital [n (%)]	4633 (53.1)	1522 (23.7)	< 0.001
In-hospital mortality [n (%)]	1116 (12.8)	2401 (38.1)	< 0.001

Data are expressed as median (Q_1, Q_3) or [n (%)]. *AAD* acute aortic dissection, *SBP* systolic blood pressure

Table S9 Univariate and multivariate logistic regression analysis for the association between in-hospital death for Stanford type A AAD and CPC accreditation

**Variable**	***OR* (95% CI)**		***P*-value**	**Adjusted *OR* (95% CI)**		***P*-value**
	**Pre-accreditation**	**Post-accreditation**		**Pre-accreditation**	**Post-accreditation**	
In-hospital mortality for Stanford type A AAD	Ref	0.624 (0.570 − 0.683)	< 0.001	Ref	0.784[a] (0.708 − 0.867)	< 0.001

[a]Adjusted for sex, age, heart rate, systolic blood pressure, surgery, comorbidities, and hospital location. *AAD* acute aortic dissection, *CPC* chest pain center, *OR* odds ratio, *CI* confidence interval, *Ref* reference

Table S10 Baseline Characteristics of Stanford type A AAD patients in the surgery group and non-surgery group before and after accreditation

Characteristics	Surgical group			Non-surgical group		
	**Pre-accreditation (n = 1179)**	**Post-accreditation (n = 7554)**	***P*-value**	**Pre-accreditation (n = 1624)**	**Post-accreditation (n = 4807)**	***P*-value**
Age [year, median (Q_1, Q_3)]	53 (45, 63)	53 (45, 62)	0.944	64 (53, 74)	62 (51, 72)	< 0.001
Sex [n (%)]						
Male	904 (76.7)	5810 (76.9)	0.857	1059 (65.2)	3330 (69.3)	0.002
Female	275 (23.3)	1744 (23.1)	0.857	565 (34.8)	1477 (30.7)	0.002
Heart rate [bpm, median (Q_1, Q_3)]	77 (66, 88)	77 (66, 88)	0.954	75.5 (64, 88)	75 (64, 88)	0.792
SBP [mmHg, median (Q_1, Q_3)]	137 (118, 160)	138 (119, 160)	0.706	140 (114, 167)	140 (116, 167)	0.663
Symptom [n (%)]						
Dyspnea	35 (3.0)	286 (3.8)	0.165	99 (6.1)	213 (4.4)	0.007
Sympathetic symptoms	71 (6.0)	605 (8.0)	0.018	108 (6.7)	413 (8.6)	0.013
Abdominal pain	117 (9.9)	505 (6.7)	< 0.001	189 (11.6)	450 (9.4)	0.008
Pain in the back or shoulders	75 (6.4)	1091 (14.4)	< 0.001	103 (6.3)	625 (13.0)	< 0.001
Toothache	12 (1.0)	112 (1.5)	0.210	27 (1.7)	97 (2.0)	0.368
Persistent chest pain	777 (65.9)	4923 (65.2)	0.623	1070 (65.9)	2965 (61.7)	0.002
Complication [n (%)]						
Malignant arrhythmia	0 (0.0)	7 (0.1)	0.296	2 (0.1)	6 (0.1)	0.987
Heart failure	1 (0.1)	10 (0.1)	0.668	13 (0.8)	22 (0.5)	0.104
Syncope	11 (0.9)	139 (1.8)	0.026	32 (2.0)	120 (2.5)	0.228
Hospital location: provincial capital [n (%)]	593 (50.3)	4040 (53.5)	0.042	295 (18.2)	1227 (25.5)	< 0.001
In-hospital mortality [n (%)]	196 (16.6)	920 (12.2)	< 0.001	660 (40.6)	1741 (36.2)	0.001

Data are expressed as median (Q_1, Q_3) or [n (%)]. *AAD* acute aortic dissection, *SBP* systolic blood pressure

Table S11 Univariate and multivariate logistic regression analysis for the association between in-hospital death for Stanford type A AAD and CPC accreditation in the surgery/non-surgery group

Variable	***OR*** **(95%CI)**		***P*-value**	**Adjusted *OR* (95%CI)**		***P*-value**
	**Pre-accreditation**	**Post-accreditation**		**Pre-accreditation**	**Post-accreditation**	
In-hospital mortality for Stanford type A AAD in the surgical group	Ref	0.696 (0.588 − 0.823)	< 0.001	Ref	0.691[a] (0.580 − 0.822)	< 0.001
In-hospital mortality for Stanford type A AAD in the non-surgical group	Ref	0.829 (0.739 − 0.931)	0.001	Ref	0.800[a] (0.706 − 0.908)	0.001

[a]Adjusted for sex, age, heart rate, systolic blood pressure, comorbidities, and hospital location. *AAD* acute aortic dissection, *CPC* chest pain center, *OR* odds ratio, *CI* confidence interval, *Ref* reference
